# Incidence of chronic kidney disease hospitalisations and mortality in Espírito Santo between 1996 to 2017

**DOI:** 10.1371/journal.pone.0224889

**Published:** 2019-11-07

**Authors:** Wesley de Souza, Luiz Carlos de Abreu, Leonardo Gomes da Silva, Italla Maria Pinheiro Bezerra

**Affiliations:** 1 Programa de Mestrado em Política Públicas e Desenvolvimento Local, Escola Superior de Ciências da Santa Casa de Misericórdia de Vitória (EMESCAM), Vitória, Espírito Santo, Brazil; 2 Laboratório de Escrita Científica, Escola Superior de Ciências da Santa Casa de Misericórdia de Vitória (EMESCAM), Vitória, Espírito Santo, Brazil; 3 Laboratório de Delineamento de Estudos e Escrita Científica, Centro Universitário Saúde ABC (CUSABC), Santo André, São Paulo, Brazil; 4 Graduate Entry Medical School, University of Limerick, Limerick, Ireland; 5 Programa de Mestrado em Ciências da Saúde da Amazônia, Bolsista CAPES Brasil, Universidade Federal do Acre, Rio Branco, Acre, Brazil; University of Mississippi Medical Center, UNITED STATES

## Abstract

**Introduction:**

Chronic kidney disease (CKD) has a set of clinical and laboratory abnormalities where renal function loss is noted. The high prevalence of comorbidity of people living with CKD, its economic impact and its prognosis have made it a public health problem, justifying the need to implement preventive measures.

**Objective:**

To analyse the mortality and incidence of hospital admissions for CKD.

**Methods:**

Ecological study with a time series design using secondary microdata of deaths and hospital admissions from patients with CKD from 1996 to 2017 in the State of Espírito Santo, Brazil.

**Results:**

The average mortality rate of CKD during the studied years was 2.92 per 100,000 inhabitants per year. During this period global mortality was a stationary phenomenon. In women, the trend of mortality from 2005 on increased 7,87% per year. Between 2008 and 2017, the average incidence hospital admissions due to CKD per year was 45.76 per 100,000 inhabitants. It was observed that the overall hospital admission increased by the equivalent of 6.23% per year. More than a half of mortality and hospitalisations correspond to male patients over 50 years of age. In terms of mortality, 32.99% corresponded to Caucasian patients, while 35.13% of hospitalisations were mixed race.

**Conclusion:**

We found that age and gender are factors associated with deaths and hospitalisations for chronic kidney disease. While hospitalisation increases 6.23% per year, global mortality remains stationary. However, from 2005 onwards a trend towards increasing of 7.87%/annual in mortality was observed in women.

## Introduction

Chronic kidney disease (CKD) is characterised by damage to the renal structure or a functional reduction of kidneys for a period of three or more months. It is a fast-growing epidemic, a major public health problem worldwide and is responsible for about 850,000 deaths a year, making it the twelfth largest cause of death [1].

This disease presents a progressive evolution and a close relationship with cardiovascular diseases, whose coexistence is associated with greater morbidity, hospitalisation events and mortality [2]. The damage to these patients also includes psychological disorders, job losses, low professional performance and low quality of life [3].

In the United States the prevalence of CKD between 2012 and 2013 reached 14% and the incidence of end stage renal disease (ESRD) was 353 cases per million/year. In Brazil, an incidence of 170 cases of ESRD per million / year and a prevalence of around 6% for CKD was reported for the same period [4].

The increase in the prevalence of CKD in the last decade is largely due to an aging population and an increase in life expectancy, accompanied by an increase in main risk factors of CKD, namely hypertension and diabetes [5].

Renal replacement therapy (RRT) is the only way to maintain life for advanced stages of the disease, the need for RRT is growing annually at a rate of 7%. The options for RRT are haemodialysis, peritoneal dialysis and renal transplantation. However, more than two-thirds of patients with CKD die without starting RRT, from those who start dialysis only one-third survive more than five years [6,7].

Kidney transplantation (living donor or deceased) confers a better likelihood of survival and improve quality of life together with lower financial costs, as compared to the other therapies. However, this is performed in only 7% of Brazilian patients [3].

According to the Brazilian Society of Nephrology (SBN) [8] in 2012, more than 100,000 people received dialysis. This is about three times more frequent in patients over 65 years old and generates huge expenses due to highly complex surgery, examinations, materials and equipment, hospitals, among others [9].

It has become a concern that the majority of patients diagnosed with CKD requiring RRT are coming from developing countries, this disease is closely related to a lack of investment in health, poverty, lack of basic sanitation, malnutrition, a high prevalence of infectious diseases and great difficulty in access to health prevention, promotion and treatment programs [10].

It has been observed an unequal access to care for patients in the early stages of CKD. During this period the disease is silent but risk factors for the progression of the disease need to be searched, identified and combated. Some factors may affect patient outcomes such as the shortage of specialist nephrologists or lack of clinician training in primary health care, as recommended by the Unified health System (SUS) [11].

Measures such as early diagnosis of renal disease, immediate referral to specialists and treatment its complications and comorbidities have been advocated as a better strategy for patient care. Such measures may reduce the short and long term mortality rate, the length of hospital stay, financial cost and consequently, improve patient´s quality of life [12].

On the other hand, there are few reliable and available registries of patients with CKD. Such resources could underpin better planning of specific actions, providing a focus to understand CKD and support the more efficient use of financial resources in Brazil [13].

In front of this scenery we wondered how are shown the mortality rate and incidence of hospitalisations for CKD in the state of Espírito Santo from 1996 to 2017?

It is believed that knowing the profile of patients, the incidence of hospitalisation and mortality rate of CKD in the state can contribute towards better disease management. It could also allow health care professionals to implement best health care practices with better administration of financial resources.

This study aims to analyse the mortality rate and hospital admission incidence for chronic kidney disease in the State of Espírito Santo from 1996 to 2017.

## Methods

This is an ecological research conducted in 2018 with time series design using secondary micro data of hospital deaths and hospitalisations for CKD patients, residents in the state of Espírito Santo [14]. This study involves only the description and analysis of population obtained from the general population census; deaths collected from the Mortality Information System (SIM). All these sources of information are in the public domain and freely accessible. No individual identification information of patients was obtained for this study.

### Sources of information systems

SIM receives, processes, gives consistency and validity to the basic cause of death recorded in the Death Certificate and provides information on more than 96% of deaths in Brazil [15].

The Hospital Information System of the Unified Health System (SIH / SUS) registers more than 85% of admissions from public and private hospitals registered in the SUS and includes 92.3% of health units in Brazil [16]. Data were collected according date of hospitalisation and from patients residing in the State of Espirito Santo.

Size of Brazilian population was obtained from the last census, carried out in 2010, and projections for the remaining years were carried out by the Brazilian Institute of Geography and Statistics website (IBGE - www.ibge.gov.br). The Espírito Santo State is located in the Southeast of the country, with a population of 3,514,952 people. It has a Human Development Index (HDI) of 0.740. In 2010 had 2,931,472 residents in urban areas and 583,480 in rural areas. [17].

Mortality rates and incidence of hospital admission and hospital mortality by CKD were analysed for the State of Espírito Santo and by Health Region (or by IBGE micro-region), crude and standardised by age group using the world standard population of World Health Organization between 2000–2025 for total population and stratified according to age group (i.e. 1 to 19 years, 20 to 49 years and 50 years or more), gender (male and female) and subtypes of Chronic Renal Insufficiency [18].

### Place and period of research

Microdata were collected by place of residence, hospital admissions and place of death. The unit of analysis was the state of Espírito Santo, with a population of 3.5 million [17]. Death data corresponded to the period between January 1, 1996 and December 31, 2016 (since 1996, death declarations were coded using ICD-10), while data from hospital admissions corresponded to the period between January 1, 2008 and December 31, 2017 (from 2008 a new set of procedures, drugs, orthotics and prosthetics and special materials were implemented by the SUS).

### Variables and data extraction

We considered all deaths and hospital admissions over one year, in public and/or private accredited hospitals of SUS during 1996–2016 and 2008–2017, respectively. Chronic Kidney Disease (CKD) was defined according to the 10th revision of the International Classification of Diseases (ICD-10), through the code used in N18.

Microdata were extracted from the file transfer service provided by the Department of Informatics of SUS (DATASUS) (www.datasus.gov.br). It is worth mentioning that these systems support the analyses of the health situation, evidence-based decision-making and public policy development in the country. Through this public official database we collected information on deaths and hospitalisations for chronic kidney disease (CKD).

The study of mortality may help to understand the epidemiology of CKD. Data obtained from information systems maintained by the Ministry of Health are reliable. Particularly in the state of Espírito Santo (Southeast Brazil), information systems have good quality and completeness [18], enabling their use as a feasible tool for assessing CKD.

The TABNET and TABWIN software were used to analyse the data. These tools were developed by DATASUS for quick tabbing on DBF files. Data were collected by two independent researchers to identify discrepancies.

### Statistical analysis

Mortality rates, incidence of hospitalisation, stratified by age groups, year by year (1996–2017), were expressed per 100,000 inhabitants, grossly and standardised using direct method, following the world standard population of WHO in the year 2000–2025 [18].

For trend analyses, we followed the methodological guidelines presented by Antunes and Cardoso [19]. We applied Prais-Winsten regression model to the population rates of deaths and hospitalisations to build time series, which allows the first-order autocorrelation to be corrected in the analysis of time series organised values. The following values were estimated: slope (β), its probability (p) and coefficient of determination (r^2^), considering a 95% significance level.

This procedure made it possible to classify trends in mortality and hospitalisation as increasing, decreasing or stationary, in addition to quantifying the annual increase degree of the rate. Stata 14.0 software was used for statistics calculations (CollegeStation, TX, 2013).

## Results

Table 1 illustrates frequencies of deaths, mortality rate (1996–2016) and the incidence and frequency of hospitalisations (2008–2017) in the state of Espírito Santo. The time trend analysis of such as variables is shown in Table 2.

**Table 1:** Mortality rates and incidence of hospital admission for CKD in Espírito Santo State, Brazil, 1996 to 2017.

STATE OF ESPÍRITO SANTO	DEATHS	%	MORTALITY RATE	HOSPITALISATIONS	%	INCIDENCE OF HOSPITAL ADMISSION
**GENDER**						
Male	1097	56,63	3,35	7918	53,86	50,27
Female	839	43,31	2,50	6704	45,61	41,39
**AGE GROUP (YEARS)**						
1–19	33	1,70	0,14	774	5,27	7,87
20–49	327	16,88	1,10	4522	30,76	29,37
50 or more	1575	81,31	13,31	9326	63,44	140,85
**RACE**						
White	639	32,99	-	5139	34,96	-
Black	203	10,48	-	1005	6,84	-
Mixed	467	24,11	-	5164	35,13	-
Asian	5	0,26	-	47	0,32	-
Indigenous	2	0,10	-	11	0,07	-
Without information	620	32,01	-	3256	22,15	-

Mortality rate and incidence of hospitalisation per 100,000 inhabitants. Source: Sistema de Informação sobre Mortalidade (SIM). Sistema de Informação Hospitalar do Sistema Único de Saúde (SIH/SUS). Data from the Department of Informatics of the Unified Health System (DATASUS - www.datasus.gov.br). Ministry of Health. Brazil.

**Table 2:** Estimates of Prais-Winsten regression analysis, related to rates of CKD, Espírito Santo, Brazil, from 1996 to 2017.

RATE OF STANDARDIZED CKD		LINEAR REGRESSION			
	Β	*P*	r^2^	APC	(CI95%)
Overall mortality	0.006	0.279	0.372	-	-0.005 : 0.017
Mortality–men	0,007	0,146	0,307	-	-0,003 : 0,016
Mortality–women	0,007	0,255	0,213	-	-0,006 : 0,020
Mortality–women > 2005	0,033	0,013	0,083	7,870	0,009 : 0,057
1–19	-0,015	0,294	0,166	-	-0,045 : 0,450
20–49	-0,020	0,007	0,290	-4,411	-0,033 : -0,006
50 years or more	0,003	0,421	0,725	-	-0,005 : 0,012
Overall hospitalisation	0,026	<0,001	0,897	6,231	0,019 : 0,033
Hospitalisation–men	0,026	<0,001	0,695	6,060	0,017 : 0,034
Hospitalisation–women	0,028	<0,001	0,931	6,550	0,019 : 0,036
1–19	0,018	0,157	-0,108	-	-0,009 : 0,045
20–49	0,015	0,004	0,344	3,625	0,006 : 0,025
50 years or more	0,029	<0,001	0,984	6,958	0,024 : 0,035

β–slope of the regression; r^2^—predictive value; CI95%—confidence interval at 95%; APC–annual percent change. Source: Sistema de Informação sobre Mortalidade (SIM). Sistema de Informação Hospitalar do Sistema Único de Saúde (SIH/SUS). Data from Department of Informatics of Unified Health System (DATASUS - www.datasus.gov.br). Ministry of Health. Brazil.

**Fig 1:**
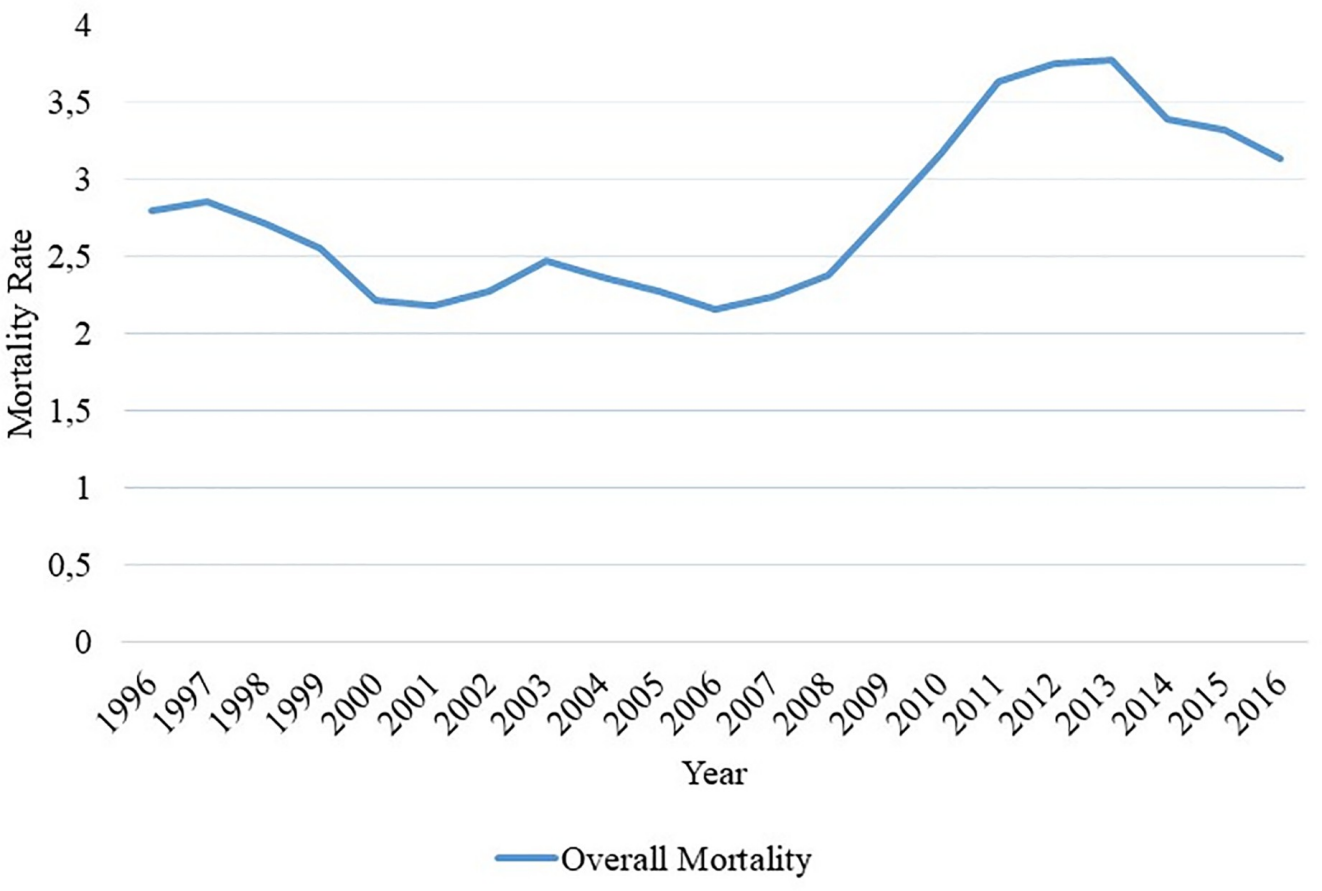
Overall mortality by CKD in Espírito Santo, Brazil, from 1996 to 2016. Source: author's own elaboration, 2018.

The State of Espírito Santo, between 1996 and 2016 reached the number of 1,936 deaths from CKD, on average 96.8 deaths per year, which corresponds to the average mortality rate of 2.92 per 100,000 inhabitants per year. It was identified that during this period, global mortality was a stationary phenomenon, as can be observed in Fig 1.

**Fig 2:**
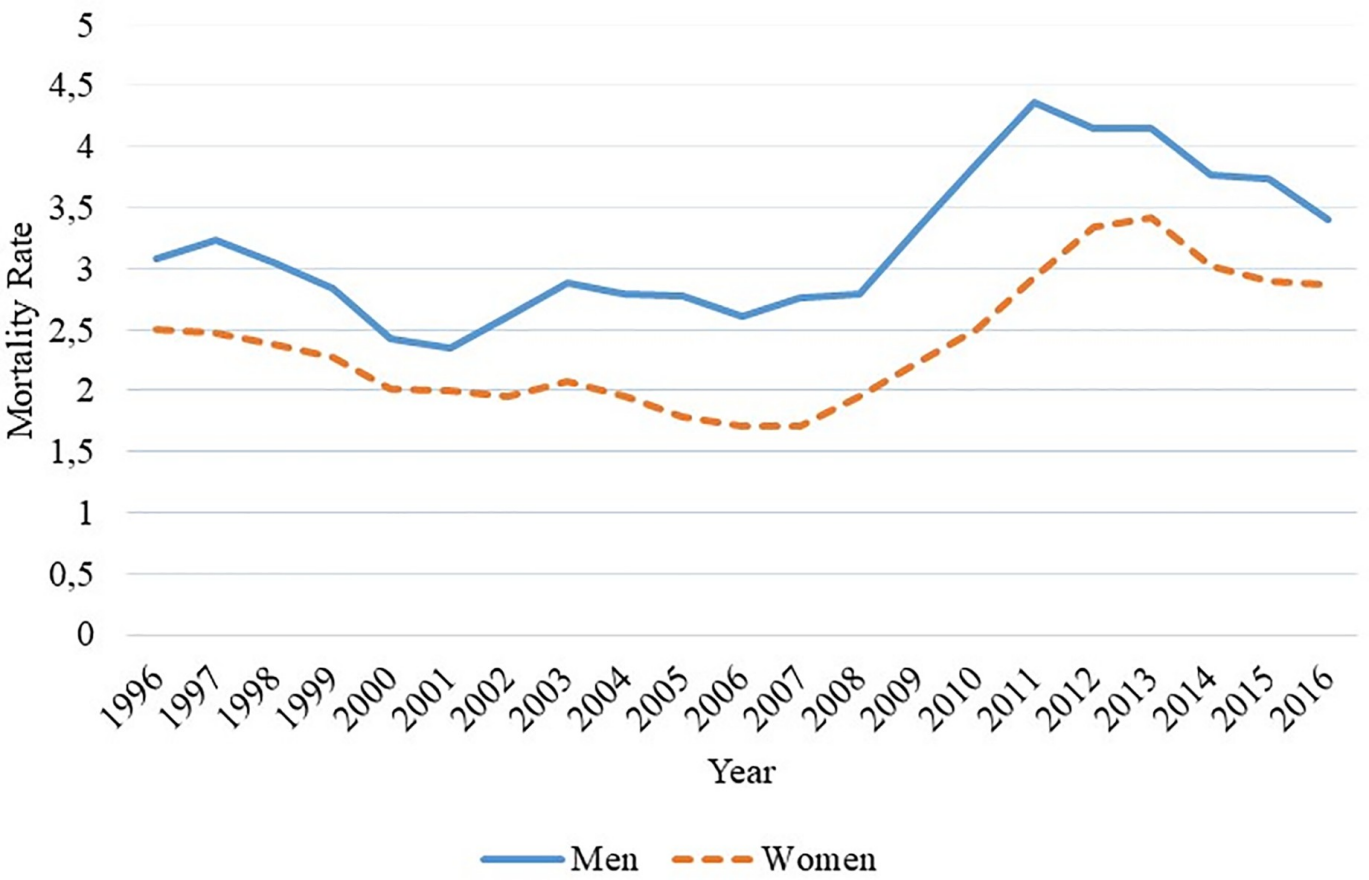
Mortality due to chronic kidney disease stratified by gender, Espírito Santo, Brazil, 1996 to 2016. Source: author's own elaboration, 2018.

When stratified by gender, the phenomenon remained stationary when evaluated over the 20-year period, although it suggests an increase between 2011 and 2014. (Fig 2). However, when evaluating the trend of mortality for years > 2005, it was observed an increase of 7.87% per year for women.

In the age groups of 1 to 19 and 50 or more years, mortality remained stationary; however, for the age group of 20 to 49 years, a decrease of 4.41% per year was noticed.

Regarding admissions, between 2008 and 2017, the average incidence per year was 45.76 per 100,000 inhabitants, which corresponds to a frequency of 14,622 cases, which is equivalent to the average of 1,624.6 hospitalisations per year.

**Fig 3:**
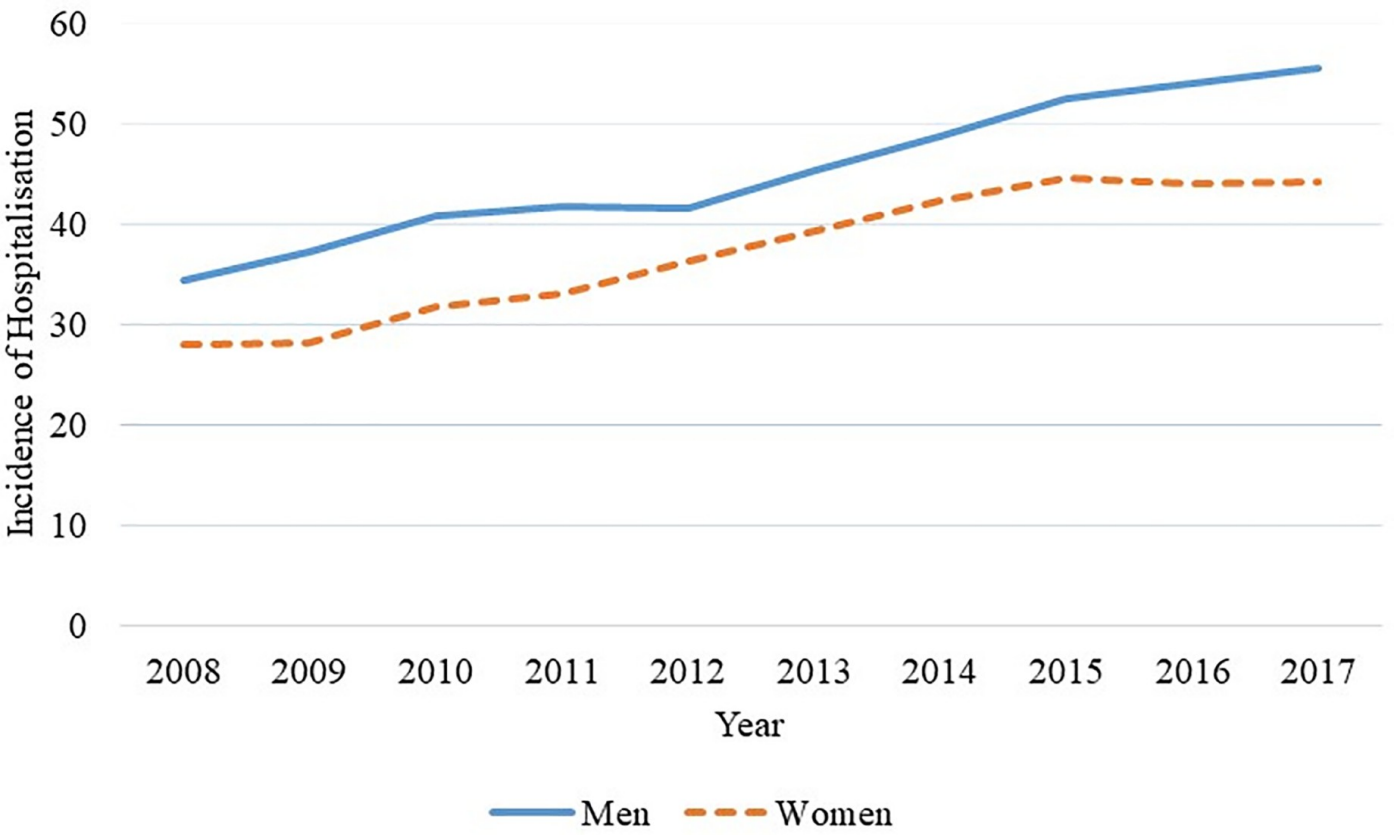
Hospitalisation for CKD stratified by gender, Espírito Santo, Brazil, from 2008 to 2017. Source: author's own elaboration, 2018.

Overall hospitalisation showed an increase of 6.23% per year. When stratified by gender, it was noted an increase in both genders, which assumes values for men and women of 6.06% and 6.60% per annum, respectively (Fig 3). Comparing hospitalisation by age group, admission was stationary for patients aged 1 to 19 years. However, in the 20–49 and 50 or more age groups there was an increase of 3.63% and 6.96% per annum, respectively.

In order to characterise the population, it was evidenced that more than a half of mortality and hospitalisation correspond to the male gender, over 50 years/old. Mortality reached 32.99% in the group of white race, while in hospitalisations 35.13% corresponds to mixed race.

## Discussion

In the state of Espírito Santo, Brazil, we found that frequency of deaths from CKD are predominant in males, aged 50 and over as well as in white people. Similarly, a study conducted in 235 patients, in Peru revealed that about six in ten deaths from CKD were in men, with a mean age of 56.4 years [20]. However, in another study with an average age of 79 (19–106), despite the divergence of the results found for gender, they found 62% of deaths were in females. The results for race converged, since most affected patients were of white race [21].

According to IBGE [22], Caucasians make up 52% of southeast region population and only 9% by mixed race, this population difference may explain the higher frequency of deaths and high hospitalisation rate in the Caucasian population. A study conducted with African-Americans showed a divergent data, observing that this population is more likely to develop kidney disease, being associated with a higher frequency of glomerulonephritis, hypertension and diabetic nephropathy [23].

Considering the relationship between diabetes, CKD and its rise with age, National Health Survey showed data 0.6% between 18 and 29 years of age; 19.9% between 65 and 74 years; and 19.6% in people older than 75 years of age [12]. In addition to the relationship between diabetes and CKD, it is noted that age may be the factor for the increase of CKD in our study as well as evidenced in the scientific literature.

Regarding gender, a meta-analysis study revealed that prevalence of CKD increased with advancing age, and two-thirds of this prevalence is in women. Prevalence has been demonstrated to be higher in more economically developed regions, besides of increasing estimates in developing countries and acceleration of population ageing [24]. This information converges with the data found in our study, where the highest incidence of both mortality and hospitalisations is related to population over 50 years.

A study in Brazil which analysed the cost of kidney disease by gender, revealed that the highest cost was for females, where the kidney disease was associated with diabetes. A higher prevalence of diabetes is found in females which, together with physiological factors inherent to female gender, result in a higher relative risk in the development of chronic renal disease and its terminal stage. It is, worth mentioning that actions are needed to strengthen the promotion, prevention, surveillance and treatment of diabetes, since it is a risk factor for CKD [25].

A systematic review and meta-analysis showed that diabetes in women boosts major adverse effects with respect to risk factors for CKD compared to men. The research also found a greater chance of ineffectiveness in the treatment when compared to men, since women are more likely to develop other diseases such as hypertension, dyslipidaemia and obesity, and it is more difficult to control glycated haemoglobin [26]. In line with this view, when mortality with stationary behaviour was stratified by gender from 2005 onwards it was noticed an increase of 7.87% per year in females. On the other hand, it was noted a higher incidence of CDK among white men linked to hypertension prevalence [27].

According to Pinheiro et al. [28], compared to men, women have more access to medical appointments, tests, and receive more medication funded by SUS. On the other hand, men seek less health care services compared to women, mainly because of job reasons [8]. Nevertheless, in the opinion of Góes and Nascimento [29], race is a factor that interferes with access to health care services observing whereas 15.4% of white women have access to services, only 7.9% of black women have the same access. Despite the increased hospitalisation for both genders in the state of Espírito Santo, over the 10 years period (from 2008 to 2017), we noted a lower incidence of hospitalisation in women compared to men. This can be justified by greater access and treatment adherence for women, suggesting that female gender is more influenced by promotion and prevention than male gender.

A Dutch study showed that during the 10 years period (from 1995 to 2005) there was an increase in mortality rate. From a total of 13,868 patients, approximately 61% died [30]. The rise in the death rate is often associated with old age and is due to emergency hospitalisations, often caused by infection inherent in the vascular access or lack of arteriovenous fistula [31].

However, this study differs from previous findings, showing that mortality in Espírito Santo is stationary over the same period of time (10 years). Although there is a correlation between hospitalisation and mortality, the discrepancy between these two modalities suggests that the health system has provided beneficial effects to this affected population, whether through promotion, prevention and/or rehabilitation, corroborating that mortality is stationary.

In São Paulo, Brazil, it was noted that during the first year of haemodialysis there were 112 hospitalisations, at a rate of 264/1000 patients. Of these, 47% died after hospitalisation [31]. Meanwhile, over a period of 10 years in Espírito Santo, there were 14,622 hospitalisations, with an annual increase of 6.23%.

Of the 63.3% of patients undergoing RRT in Taiwan, 33.7% died within 60 days after hospital discharge [32]. A study conducted in the administrative region of Brittany, France, showed worse access to treatment of CKD and higher mortality among patients living in socioeconomically disadvantaged areas [33]. Also, in Argentina, a close relationship was observed between low income, low educational level and unemployment, with a higher mortality in patients on peritoneal dialysis [34]. On the other hand, higher educational levels in developed countries relates to lower morbidity and mortality rates of CKD and RRT [35].

Data from Brazilian Society of Nephrology (SBN) revealed the existence of 747 dialysis centres operating in Brazil. Of this total, 49% are located in the Southeast region, which in turn includes Espírito Santo. Nephrology corresponds to about 1% of medical specialties, and 22% of nephrologists are located in the Southeast region [8].

According to the Brazilian Registry of Transplant (RBT), the number of transplants performed in Brazil is still far to fill country needs. In the Southeast of the country, in the period from January to September 2018, 43 kidney transplants were performed [36]. Based on these data from specialised services, it is suggested that the increase in transplants has promoted an increase in hospitalisation and, thus, controlling mortality and benefiting the population.

In countries where the health system is unable to cover the cost of dialysis, prevention is a key resource that can save lives. Thus, quality care regarding screening, diagnosis, treatment and the cost-effectiveness of early intervention needs to be transformed into data. Health Data is essential, it will allow corroboration with transparency about the economic consequences of kidney disease and the need to recognize the importance of clinical guidelines [37].

In a study carried out in Beijing, the authors showed that diabetes, hypertension and heart disease are the most common comorbidities in health care centres. However, in view of the chronic problems, a program implementation tool was developed focusing on patients, where a success factor has been the collaboration between doctors and patients. The management system of these diseases is monitored in real time, with an early warning response to ensure early diagnosis and intervention [38].

With real-time monitoring, family physicians are notified when is a delay in patient follow-up, which ensures the ongoing management of the individual's health. In addition, data are generated in the system, which enables the health professionals to track the results of short-term interventions, identify other health issues and adjust the management protocol. It was confirmed that the application of intelligent system management of chronic diseases has provided significant improvements in the condition of patients. The authors, therefore, argue that this should be promoted in China as well as in other developing countries [38].

Countries have begun to develop strategies for the prevention and control of non- communicable diseases due to its increasing rates of death and disability, but renal disease has low visibility even being part of this group. Some countries do not particularly address renal disease, showing a lack of awareness about other related, non-communicable diseases, such as diabetes, hypertension and obesity, which can lead to kidney disease as a secondary outcome [39,40].

Local statistics, where available, emphasise the importance of expressing the burdens as well as the associated financial impact of medical care and disability. In addition, for proposals for kidney disease to be accepted, these should be articulated together with existing or planned public health initiatives, and include other parties, such as target population that will benefit, governments and potential allies [40].

In Brazil, the Ministry of Health finances most treatments of CKD and end stage disease. Therefore, there is an urgent need for strategies that support the screening of kidney disease, especially in populations of higher risks. This will help to focus public policies on reducing the growth of incidence of this disease, since this condition seriously impacts on the budget of public health system [25].

Research is imperative to find new ways to achieve an optimised health system and meet the medical needs of researchers, managers and patients. This will help to promote improvements in the health system users through knowledge, new diagnosis or treatments [41]. In this regard, evaluations of users' satisfaction with services are common. However, it is noted that when the evaluation is applied to service providers, it is still a sensitive topic in Brazil and in the rest of the world [42].

It is clear that CKD is a serious public health problem that needs to be addressed and recognised by health professionals so they can provide a comprehensive care to patients ranging from prevention to rehabilitation; highlighting the importance of preventive measures and promoting the health through referring to risk factors and also key factors related to treatment adherence.

It is relevant the need to expand the discussion presented in this study as epidemiological information of the state of Espírito Santo, so that health professionals can establish decisions that meet the needs of the population.

It is expected that our findings may contribute to a greater visibility of the incidence of hospitalisations and mortality due to CKD. Although this study represents only one Brazilian state, it has pertinent results to incite the creation of protocols for treatment and the prevention of injuries, considering the profile of this population.

Mortality studies have their own limitations when secondary data are analyzed, especially regarding the validity of identifying the underlying cause of death. However, as was previously mentioned, the data used in this study, even containing possible underreporting is the oficial source from the Ministry of Health of Brazil available for decision making and formulation of public policies. Thus, unexpected events can occur at any time, as it involves different variables, which may result in additional risk of error.

## Conclusion

By analyzing the incidence of hospitalisations and mortality, we found that age and gender are factors associated with deaths and hospitalisations for chronic kidney disease. While hospitalisation increases 6.23% per year, global mortality remains stationary. From 2005 onwards a trend towards increasing of 7.87%/annual in mortality was observed in women.

## Supporting information

S1 File Data reporting. (XLSX)
